# Temperature Effects on the Compressive Behaviors of Closed-Cell Copper Foams Prepared by Powder Metallurgy

**DOI:** 10.3390/ma14216405

**Published:** 2021-10-26

**Authors:** Bin Han, Yunyu Li, Zeyu Wang, Xi Gu, Qi Zhang

**Affiliations:** School of Mechanical Engineering, Xi’an Jiaotong University, Xi’an 710049, China; xjtu_liyunyu@foxmail.com (Y.L.); Wangzeyu00@Yahoo.com (Z.W.); xjtu_guxi@mail.xjjtu.edu.cn (X.G.)

**Keywords:** closed-cell copper foam (CCCF), graded pore, uniform pore, high temperature

## Abstract

A fabrication technology of closed-cell copper foams (CCCFs) based on powder metallurgy is proposed, by using the expanded polystyrene foams (EPS) spheres with the prescribed diameter as the space holder before sintering. The material characterization and the quasi-static compressive behaviors of both uniform and graded CCCFs at different temperatures were experimentally studied. A high temperature weakens the initial compressive modulus, plateau stress, and effective energy absorption for both uniform and graded CCCFs; meanwhile, the onset strain of densification and the maximum energy absorption efficiency are less sensitive to temperature, especially for the graded CCCFs. Compared with the uniform CCCF, the graded CCCF with even a small relative density exhibits superiority in terms of the effective energy absorption and the maximum energy absorption efficiency, attributed to the much larger onset strain of densification for the gradient pore arrangement. Finite element simulations based on the ideal sphere foam model can basically mimic the compressive performance of the CCCF samples. It is also found that both the decrease of pore diameter and the increase of cell wall thickness could improve the compressive performance of the CCCFs.

## 1. Introduction

Porous metals or metal foams exhibit great performance in energy absorption, electrical conductivity, and thermal conductivity [1,2,3,4,5], and are widely applied as crashworthiness components, battery electrodes, and heat exchangers [6,7,8,9,10,11]. Currently, metal foams are usually fabricated by electro-deposition [12], casting or powder metallurgy [13,14], and additive manufacturing [15,16,17]. Electro-deposition [18] is also applied to produce open-cell metal foams, in which the metal is deposited onto the surface of the organic support by an electrochemical method, and the support is subsequently removed by degreasing. Using a deposition process to prepare metal foam is complicated, with low production efficiency and a high cost. Casting is a commonly used method to fabricate closed-cell metal foams, which can be divided into three categories. The first is the direct injection of gas to form pores [19]. The second is that the foaming agent is put into the molten metal, and pores are formed through the release of the gas by the foaming agent. Finally, the third is that the metal liquid is poured into the solid inorganic material, and the inorganic material is later removed to form the pores [20]. The production of metal foam by casting is easy to realize industrial production and the cost is low, but it also has some shortcomings, e.g., uncontrollable cell size, cell shape irregularity, non-uniform distribution of the cells, inhomogeneous composition of the matrix material, and introduction of impurities [21]. As for the powder metallurgy (PM), the metal powder is sintered together with space holders, in which metal powder is used to form a metal matrix, while the space holders are employed to form the closed or open cells [22]. PM can be used to produce closed-cell and open-cell metal foams with a controllable cell shape and cell size, uniform distribution of the cells, and the necessary composition and properties of the metal matrix [23]. Recently, additive manufacturing is also employed, but only to fabricate open-cell foams by binder jetting [15] or a 3D-printed polymer mold [16,17], followed by pressureless sintering.

Closed-cell foams, with foam cells separated by the solid faces, possess better stiffness/strength and energy absorption than open-cell foams with the voids interconnected and not having cell faces. The closed-cell foams are found to be suitable for energy absorption, blast resistance, foam core sandwich panels, foam-filled tubes, sound and noise attenuation, dampers, etc. [24,25]. Defects (e.g., curved cell wall, corrugation, cell shape irregularity, missing cell wall, non-uniform distribution of cell wall thickness, etc.) are commonly found for closed-cell foams made by the traditional casting method (i.e., liquid metallurgy route) [14], which greatly weakens the mechanical performance [22,26]. Moreover, the closed-cell foams with graded foam pores could outperform the uniform foams in terms of mechanical and energy absorption capacity [27,28]. To reduce defects and obtain the controllable cell shape and cell size, composite metal foams (CMF) with closed cells are proposed, which are made of closely packed metallic hollow spheres or a cenosphere with a metallic matrix that fills the empty spaces in between spheres [29]. Rabiei et al. [30,31,32] employed steel spheres to fabricate various CMFs by casting and PM, studied their mechanical properties, and found that these CMFs displayed superior compressive strengths and energy absorption capabilities. Skolianos et al. [33] fabricated aluminum-cenospheres syntactic foams with different compositions and varying relative densities by PM, and investigated their physical and mechanical properties and the influence of the PM route on the deformed refined mechanisms and fracture strength. Mondal et al. [34] used a cenosphere as a space holder to make Ti-cenosphere syntactic foam through PM route, and studied the effect of the varied cold compaction pressure on the density, cenosphere crushing, and strength of the Ti-cenosphere foam. The as-fabricated composite metal foams contain two or more materials, of which the excess impurities might weaken the performance of the metal foam in terms of electrical or heat conductivity.

In this paper, a novel preparation method based on PM is developed to fabricate closed-cell copper foams (CCCFs), in which the expanded polystyrene foam (EPS) spheres with a prescribed diameter are used as space holders, and copper is the matrix metal. There is almost pure copper in the as-fabricated foam sample, which would have better electrical conductivity and thermal conductivity than the CMF, and better mechanical performance than the open-cell copper foam [35,36,37]. Compared with the closed-cell aluminum foams [38,39], the closed-cell copper foams have a higher temperature tolerance, so they are suitable for the energy absorption materials in the high temperature environment. This fabrication process has the advantages of a low production cost and short fabrication time, and the CCCFs fabricated by this process have the advantages of an adjustable pore structure, controllable porosity, but less pore defects. The graded foam with better energy absorption can also be directly prepared by adjusting the size of the EPS sphere without secondary processing.

In this work, the new preparation method of closed-cell copper foam was firstly introduced. The as-fabricated CCCFs were characterized in terms of density, element, and macro- and micro-structural characteristics. The mechanical properties of the sintered uniform and graded foam samples, including compression capacity and energy absorption efficiency at different temperatures were experimentally investigated to evaluate the mechanical performance of CCCFs in a high temperature environment. Finite element (FE) simulations based on the sphere foam model were employed to explore the deformation characteristics of the uniform and graded CCCFs. In addition, the effects of pore diameter, wall thickness, and pore arrangement on the compressive performance of CCCFs were discussed.

## 2. Materials and Methods

### 2.1. Materials

As the matrix material, the copper powder (commercially available) used in the experiment was 99.6% pure and the powder size was 100 mesh. Before using, the copper powder needed to be annealed at 300 °C in a reductive atmosphere to stabilize the crystal structure of the powder. EPS spheres were used as a space holder, and their diameters were 3–5 mm. The spheres were put into anhydrous ethanol to remove the impurities on their surfaces. The adhesive is a kind of glue made of polyvinyl alcohol and water at a ratio of 1:1.

### 2.2. Fabrication Technology

**Figure 1:**
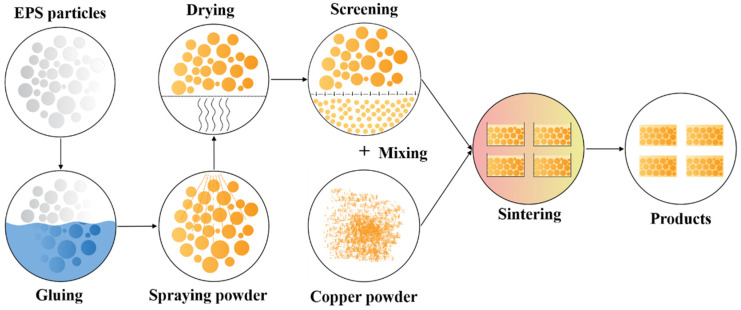
Schematic diagram of material fabrication process.

The fabrication process (shown in Figure 1) of CCCFs consisted of five steps:

(1) Immerse the EPS spheres (treated with anhydrous ethanol) into the vinyl glue (diluted with water with the same volume), covering the surface of all of the EPS spheres with the glue.
(2) Take out the glue-covered EPS spheres and put them on the absorbent cotton for 5 min to remove the excess liquid, then evenly spray a layer of copper powder on the surfaces of the EPS spheres.
(3) Dehydrate the EPS spheres covered with copper powder and glue in N_2_ for 4 h at the temperature of 80 °C.
(4) Screen out some balls with specific diameters, and mix the copper powder and the EPS spheres with the prescribed diameter in the crucible, layer by layer. Through this way of screening and mixing, the morphology and stability of the pores can be well controlled. This is the critical step which dominates the specified pore distribution, which determined either uniform or graded closed cells in the copper foam.
(5) Put the crucible into a tubular furnace to remove the EPS spheres in N_2_, with the sintering temperature at 850 °C and the time at 30 min. This is a loose sintering process, which enables the compactness of the pore wall without cold compaction. The EPS spheres disappeared after the sintering, leaving only the prescribed sphere pores in the copper foam block.

Through the above process, both uniform and graded closed-cell copper foams with specified pore diameters can be fabricated.

### 2.3. Material Characterization and Compression Tests

The density of the fabricated samples was calculated as per Archimedes principle. Further, relative density was calculated with respect to the sold copper density (8.9 g/cm^3^). The metallographic structure and the microscopic feather of the cell walls in the copper foams were separately analyzed through the optical microscope (Nikon Lv150, Nikon, Tokyo, Japan) and the scanning electron microscope (FEI Quanta FEG 250, Zeiss, Jena, Germany). The X-ray fluorescence spectrum (XRF) analysis was carried out to evaluate the composition of the as-fabricated foam samples. To obtain the material properties of the copper matrix at different temperatures, the copper blocks without any foam cells were fabricated by pressureless and EPS-free powder metallurgy with the same sintering temperature and sintering time as the copper foam.

Both the copper blocks and CCCFs were cut into specified compressive samples by the wire-cut electric discharge machine. Compression experiments of copper blocks and CCCFs at specified temperatures were conducted by using the INSTRON 5982 material testing machine equipped with a high temperature furnace. Especially for the copper foams, the ratio of the load applied to the samples and the cross-sectional area was called the nominal stress, which is denoted as *σ*. The ratio of the variation Δ*L* in the height direction of the samples to the original length *L* was called the nominal strain, which is denoted as *ε*, and the *σ*-*ε* curve could be obtained. The compressive experiment was conducted at a specified temperature with a constant strain rate of less than 10^−3^ s^−1^.

Two types of CCCFs samples were fabricated for the compression tests. One type of sample had the uniform pores with the pore diameters of about 5 mm. The other type of sample had the graded pores, and the pore diameters were arranged in size of 5 mm, 4 mm, and 3 mm along the gradient direction. Each type of sample was tested under the three different temperatures of 25 °C, 300 °C, and 500 °C. Three samples are used for each test to ensure the repeatability of the results, with the compressive stress–strain curve presented as the average of the test results. All tests were carried out quasi-statically with a nominal displacement rate of 0.5 mm/min.

### 2.4. FE Modelling

**Figure 2:**
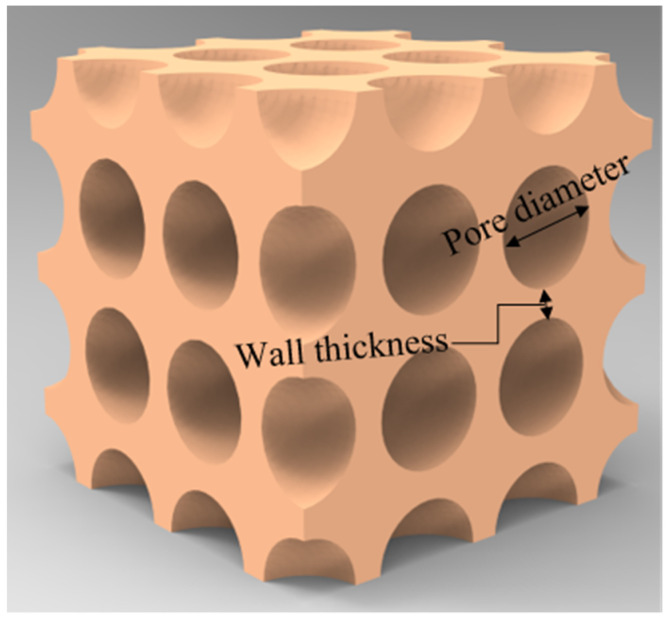
Schematic of the sphere foam model for the closed-cell copper foam.

The commercially available FE code ABAQUS/EXPLICIT was utilized to numerically explore the deformation characteristics of the uniform and graded CCCFs, based on the sphere foam model as sketched in Figure 2. The effects of pore diameters, wall thicknesses, and pore arrangement on the compressive performance of CCCFs were also numerically investigated. All of these CCCF models were set to be cubes of 15 mm × 15 mm × 15 mm. To improve the calculation accuracy, the foam models were meshed by the second-order modified tetrahedral elements C3D10M with an element size of about 0.5 mm, which could accurately capture the contact deformation for the large deformation of the foam. A mesh sensitivity study was carried out to confirm that the current mesh style was able to achieve high numerical accuracy and low computational cost simultaneously. In the finite element model, general contact was used between all of the contacting surfaces with the Coulomb friction coefficient fixed at 0.2. The copper foam model was compressed between the two rigid pressure plates, of which the bottom plate was fixed, and the top plate was moved along the compressive direction at a sufficiently slow speed of 1 m/s. It is valid that the kinetic energy in the whole model was quite small compared with the plastic dissipation during the compressive process, suggesting that the loading process in the simulation could be treated as the quasi-static crushing process. The parent material was modeled as an isotropic solid and obeyed the von Mises J2-flow theory, with the temperature-dependent material parameters obtained from the quasi-static compression stress versus strain curves at different temperatures. The copper was assumed sufficiently ductile to sustain large strains without fracture, which was confirmed by the compression tests of the copper block.

## 3. Results and Discussion

### 3.1. Microstructures and Components

**Figure 3:**
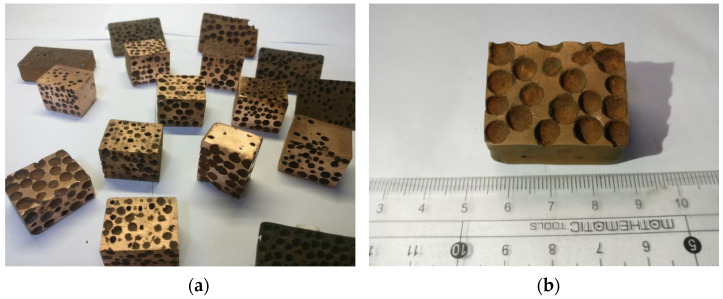

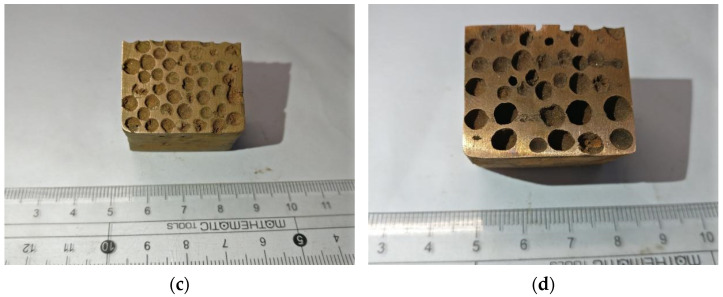
Photos of several closed-cell copper foam (CCCF) samples: (**a**) all kinds of CCCF samples; (**b**) uniform CCCF with pore diameter of 5 mm; (**c**) uniform CCCF with pore diameter of 3 mm; (**d**) graded CCCF with a gradient arrangement of pore diameter (3-4-5 mm).

**Figure 4:**
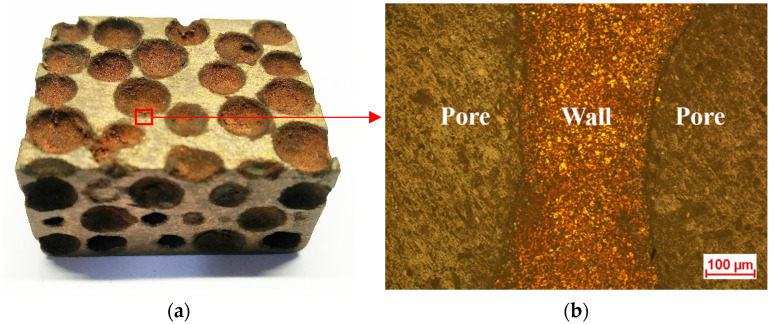
Microphotograph of the pore wall: (**a**) produced CCCF sample; (**b**) cell structure schematic diagram of CCCF sample.

Macroscopic photos of the as-fabricated CCCFs with uniform pores and graded pores are shown in Figure 3, with the microphotograph of the pore wall as presented in Figure 4. The prescribed diameter of the sphere pores in the present study ranges from 3 mm to 5 mm. It can be found that the sphere pores with equal diameters are approximately evenly distributed for the uniform CCCFs (see Figure 3b,c); meanwhile, for the gradient CCCF (Figure 3d), the sphere pores with different diameters are arranged in a prescribed gradient direction. As shown in Figure 4, the smooth and complete cell wall of the sphere pore can be observed in the produced sample, which implies that the foam cell structure made from the powder did not collapse in the process of EPS decomposition. The complete geometry of the sphere pores is preserved in the foam samples after the sintering, with less meso defects which are commonly found in the aluminum closed-cell foams [22,26,40]. 

**Figure 5:**
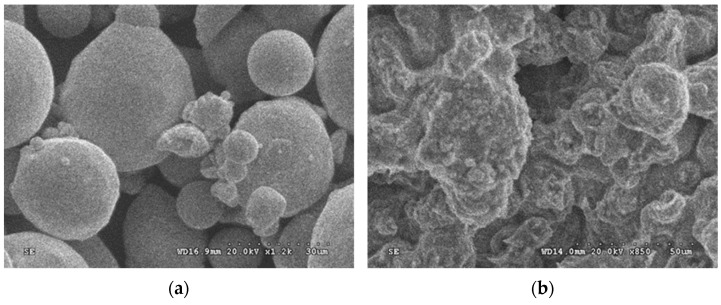

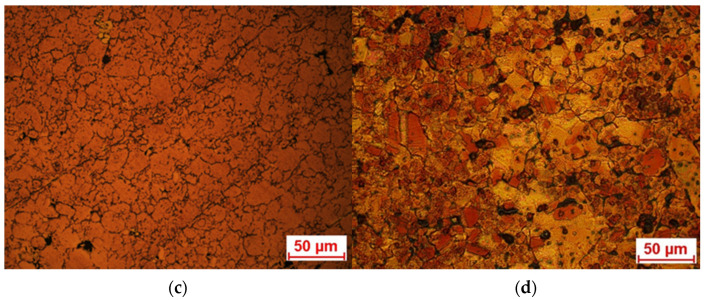
Scanning electron microscope micrograph of the metal matrix, of which the slice was cut from the copper foam (**a**) before and (**b**) after sintering; metallographic photos (**c**) before and (**d**) after corrosion of metal matrix.

A comparison of the scanning electron microscope micrograph of both the copper powder before PM and the copper slice after PM (as shown in Figure 5a,b) indicates that the copper powder and the metallic copper powder were well bonded together at the sintering temperature of 850 °C within 30 min. Figure 5c,d present the metallographic photos before and after corrosion of the metal matrix. It can be observed that quite a few grains are approximately spherical in shape, and the grain size is below 50 microns. It is also found that there are some micro-voids with a size less than 20 μm in the metal matrix (see Figure 5d), which is inevitable and unavoidable in the pressureless sintering process. 

**Figure 6:**
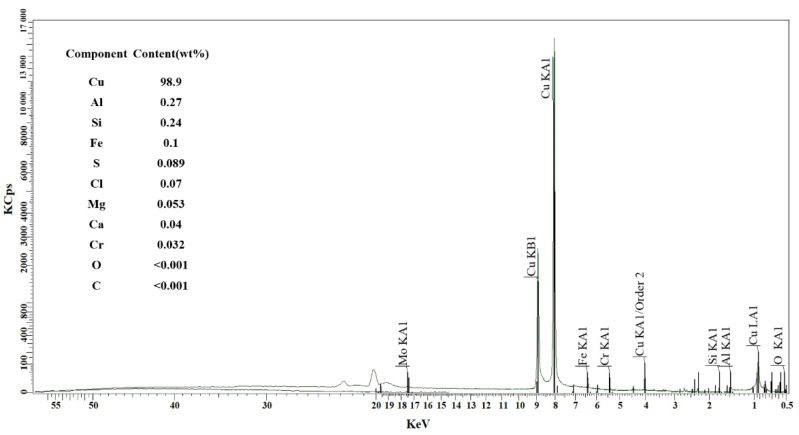
XRF spectral line diagram of the sintered sample.

**Figure 7:**
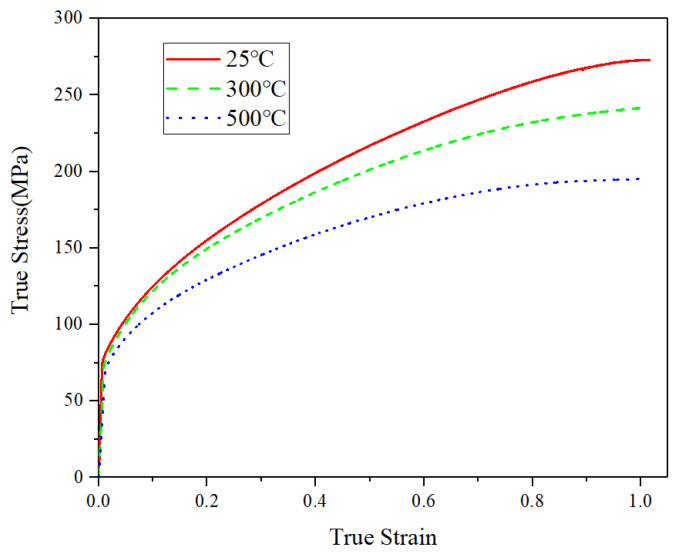
The compressive stress–strain curve of the copper block at different temperatures.

Figure 6 shows the XRF spectral line diagram of the sintered sample, in which the main elements and their contents can be obtained. It can be clearly seen that the intensity of the main peak of the copper element is very high, the intensity of the second peak of the copper element is higher than that of the main peak of the oxygen element, and the data shows that the element content of copper accounts for 98.9% in the total elements. This indicates that there is less chemical residue of EPS in the copper foams, and no oxidation phenomenon in the sintering process occurred under the condition of nitrogen protection, which will help ensure the functional properties of the copper matrix. Moreover, the compressive stress–strain curves of the copper block at different temperatures as presented in Figure 7 demonstrate that the copper metal after sintering shows good mechanical properties and ductility.

### 3.2. Compressive Performance

**Figure 8:**
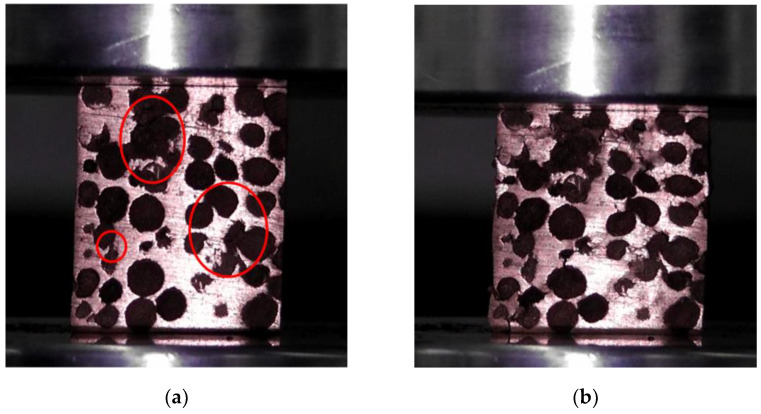

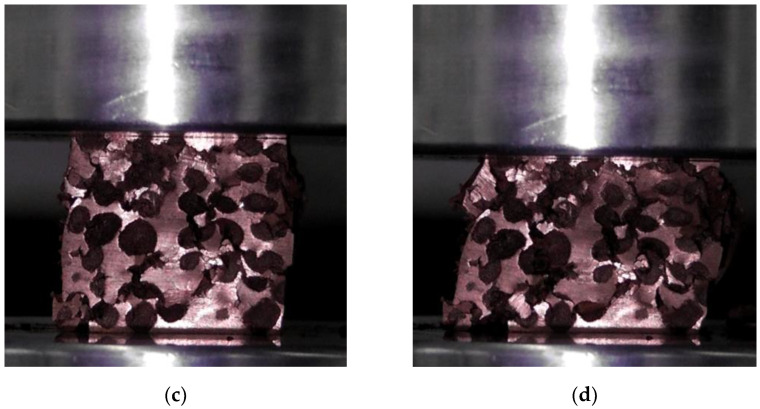
Deformation snapshots of the uniform CCCF with a pore diameter of 5 mm in the compression process. Some initial damages induced by the wire-cut electric discharge machining are marked by the red circles in (**a**). (**a**) *ε* = 0; (**b**) *ε* = 0.1; (**c**) *ε* = 0.2; (**d**) *ε* = 0.3.

**Figure 9:**
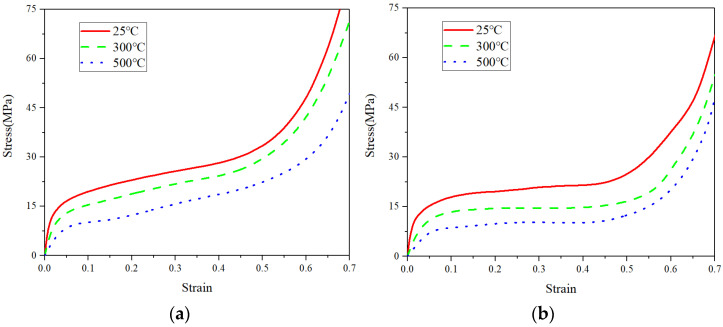
Stress–strain curves of copper foams with (**a**) uniform pores and (**b**) graded pores at different temperatures. The relative densities of the uniform and graded copper foams are 0.42 and 0.37, respectively.

The deformation photos of the uniform CCCF sample with the pore diameter of 5 mm, in the compression process at the ambient temperature of 25 °C, are shown in Figure 8, with the compressive strain changing from 0 to 0.3. Before compression, it is observed that some initial damage to the cell walls exist on the surfaces of the foam samples (see the circle region of Figure 8a), which are induced by the wire-cut electric discharge machining. Such cutting-induced damages cause the subsequent fracture failure for some of the cell walls at a large compressive strain. However, most of the cell walls collapse in the form of plastic deformation layer by layer in the compressive process, which corresponds to the smooth compressive stress–strain curves and the long plateau stage, as shown in Figure 9a. Attributed to the good ductility of the copper matrix, no fracture fragments can be observed, even for the compressed CCCF samples after the densification.

The measured compressive stress–strain curves of both uniform and graded CCCFs at different temperatures are presented in Figure 9. The compressive response of all of the samples undergoes three stages in the loading process. The first stage is elastic deformation; the deformation in this stage is mainly dominated by the elastic bending deformation of the cell wall. The second stage is plastic deformation, where a long plateau stage is formed in the stress–strain curves. The deformation of the CCCFs in this stage is mainly bending, folding, and some fractures of the cell walls, and this stage contributes most to the energy absorption. The third stage is the densification of the CCCFs, in which CCCFs lose their good energy absorption capacity due to the compaction of most cell walls. It can be clearly seen that the bearing capacity of either uniform or graded CCCFs decreases with the increase of temperature. It is interesting and noteworthy to compare Figure 9a with Figure 9b, where the stress plateau stage of the graded CCCF is much more obvious and longer than that of the uniform CCCF. This implies that the graded CCCFs might exhibit a better energy absorption efficiency than the uniform CCCFs, which would be confirmed by the later comparison of the extracted data as listed in Table 1. For the uniform CCCFs, even in the approximate plateau stage, the stress increases faintly with the compressive strain.

**Figure 10:**
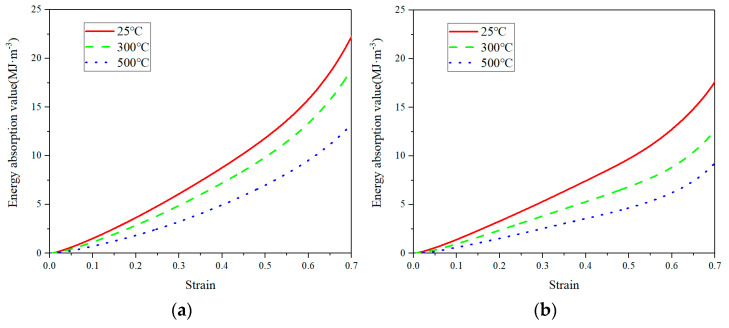
Curves of energy absorption per unit volume of CCCFs with (**a**) uniform pores and (**b**) graded pores at the different temperatures, as a function of the compressive strain ε.

From the stress–strain curves of Figure 9, the energy absorption of CCCFs can be calculated by [2,41]:

(1) $$W=\int_{0}^{\varepsilon }\sigma d\varepsilon$$

where *W* denotes the amount of energy absorbed per unit volume of copper foams, in the unit of $\mathrm{MJ}\cdot m^{-3}$; and σ denotes the stress value at compressive strain ε. The curves of energy absorption per unit volume as the function of ε are given in Figure 10. Because the energy absorption of CCCFs occurs mainly in the second stage of compression, the energy absorption in this stage is almost linear with the increased strain, which corresponds to the stress plateau stage.

**Figure 11:**
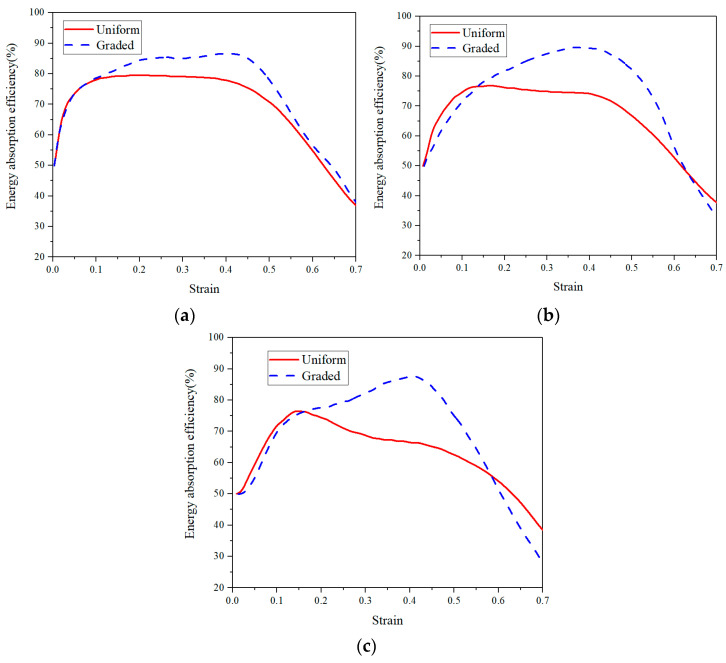
Energy absorption efficiency versus compressive strain curves of uniform and graded CCCFS at the temperature of (**a**) 25 °C, (**b**) 300 °C, and (**c**) 500 °C.

Another important indicator to evaluate the energy absorption capacity is the energy absorption efficiency, which is defined as the ratio of the actual energy absorption value and the ideal energy absorption value, calculated by [2,41]:

(2) $$\eta =\frac{\int_{0}^{\varepsilon }\sigma d\varepsilon }{\sigma_{\max}\varepsilon }$$

where η denotes the energy absorption efficiency, and $\sigma_{\max}$ is the maximum stress value within the strain range of 0 ~ ε. From the η − ε curves as shown in Figure 11, it can be found that the energy absorption efficiency of the graded CCCF is obviously larger than that of the uniform CCCFs, especially in the second stage of the compression. The maximum energy absorption efficiencies $\eta_{\max}$ of the graded CCCF at different temperatures are all beyond 80%, while those of the uniform CCCF never exceeds 80%. The highest maximum energy absorption efficiency appears for the graded CCCF compressed at the temperature of 300 °C, up to nearly 90%.

The onset strain of densification $\varepsilon_{d}$ at which $\eta_{\max}$ is reached, the initial compressive modulus, plateau stress, effective energy absorption $W_{e}$ (i.e., $W_{e}=\int_{0}^{\varepsilon_{d}}\sigma d\varepsilon$), and the maximum energy absorption efficiency $\eta_{\max}$ of both uniform and graded CCCFs at different temperatures are all listed in Table 1. The uniform CCCF with a larger relative density is superior to the graded CCCF with a small relative density, in terms of both the initial compressive modulus and plateau stress. However, the effective energy absorption $W_{e}$ and the maximum energy absorption efficiency $\eta_{\max}$ of the graded CCCF are both larger than those of the uniform CCCF, attributed to the much larger onset strain of densification $\varepsilon_{d}$ for the foam with gradient pore arrangement. Moreover, the high temperature weakens the initial compressive modulus, plateau stress, and effective energy absorption for both uniform and graded CCCFs. Nevertheless, the onset strain of densification $\varepsilon_{d}$ and the maximum energy absorption efficiency $\eta_{\max}$ are less sensitive to the temperature, especially for the graded CCCFs.

### 3.3. FE Analysis

The main purpose of the current simulation is to explore the plastic deformation characteristics of the internal foam cell walls (i.e., the collapse of the foam pore), which cannot be observed through the apparent failure evolution of the surface profile of the compressed CCCF specimen in the experiments. Moreover, some parametric study could be numerically explored in the later discussion. 

Although there exist some defects (i.e., the micropores induced from the sintering and surface damages by cutting) for the fabricated CCCF specimens, only mesoscopic sphere foam pores are constructed in the FE foam models. To explore the influence of manufacturing defects on the compressive performance, the multi-scale numerical method would be probably needed, which might cost too much computations [42,43]. At present, the multi-scale simulation of metal foams still has great challenges. Published work [44,45] implies that this method is only limited to 2D simplified models or 3D simulation calculations for models with very small size. At the same time, complex simulation pre-processing and sample scan reconstruction are required [26,46]. For brevity, only the mesoscopic pore model is employed for the foam model in the present study.

#### 3.3.1. Validation against Experimental Results

**Figure 12:**
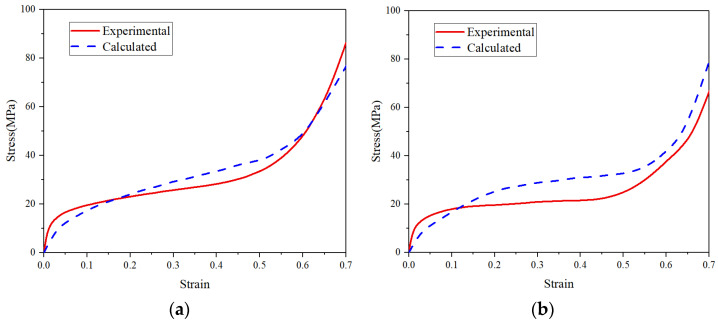
Comparison of experimental and calculated curves of the closed-cell copper foams with (**a**) uniform pores and (**b**) graded pores at 25 °C.

**Figure 13:**
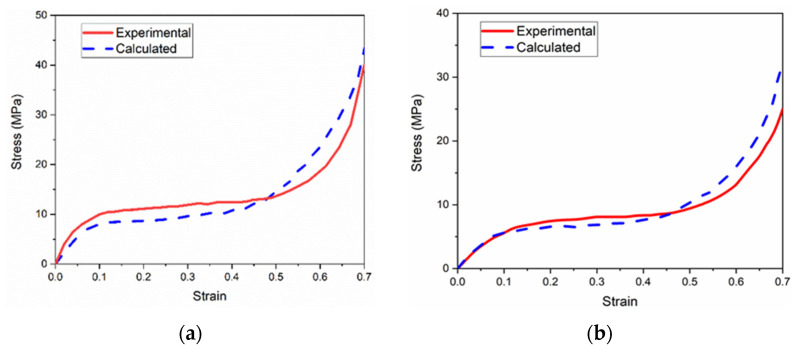
Comparison of experimental and calculated curves of the uniform closed-cell copper foams at (**a**) 300 °C and (**b**) 500 °C.

Figure 12 compares the experimental and calculated stress–strain curves of both the uniform and graded CCCFs compressed at 25 °C; Figure 13 compares the experimental and calculated curves of the uniform CCCF at both 300 °C and 500 °C. The approximate coincidence of the measured and calculated compressive stress–strain curves implies a roughly good agreement between simulations and experiments of the CCCFs. This also implies that the present FE models based on the ideal sphere foam model could basically mimic the compressive performance of the CCCF samples. However, some deviation exists between the simulations and the tests. The deviation might be attributed to the ignorance of material fracture, faint uncertainty of foam pore distribution, micro-defects, and initial surface damages, which could be possibly considered in a sophisticated multiscale numerical method in our future work. Similarly, the material fracture damage can be considered in the future work.

#### 3.3.2. Parametric Study

**Figure 14:**
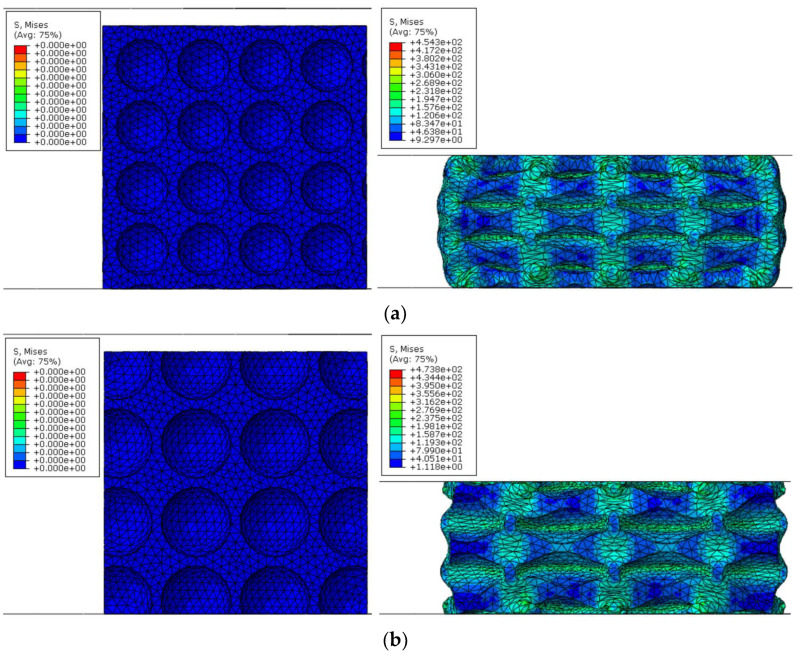

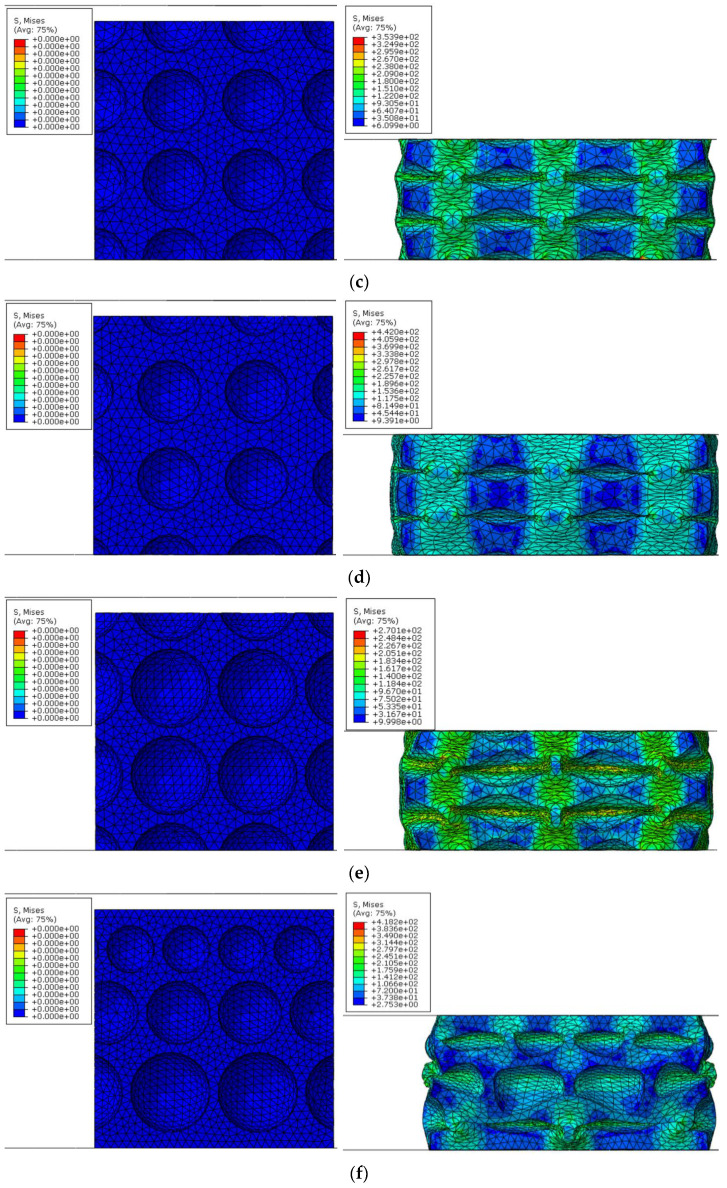
The deformation configuration pictures of different CCCF models at the compressive strain of zero (**left**) and 0.5 (**right**). (**a**) *D* (pore diameter) = 3 mm, *t* (wall thickness) = 0.5 mm. (**b**) *D* = 4 mm, *t* = 0.5 mm. (**c**) *D* = 4 mm, *t* = 1.0 mm. (**d**) *D* = 4 mm, *t* = 1.5 mm. (**e**) *D* = 5 mm, *t* = 0.5 mm. (**f**) Graded, *t* = 0.5 mm, with pore diameter *D* ranging from 3 mm to 5 mm.

**Figure 15:**
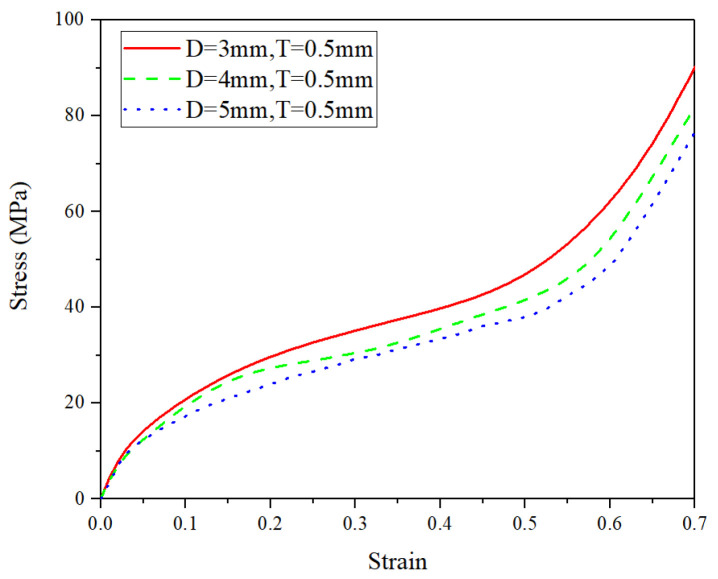
Stress–strain curves of the uniform CCCFs with the same wall thickness but a different pore diameter (*D* = 3 mm, 4 mm, and 5 mm).

**Figure 16:**
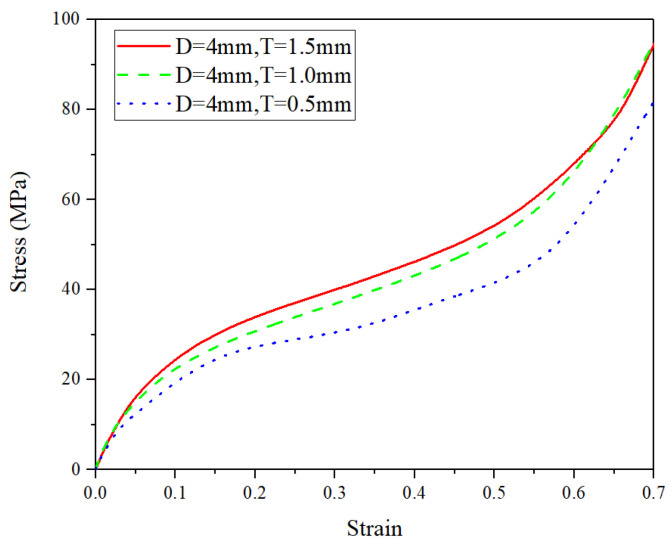
Stress–strain curves of the uniform CCCFs with the same pore diameter but a different wall thickness (*t* = 0.5 mm, 1 mm, and 1.5 mm).

**Figure 17:**
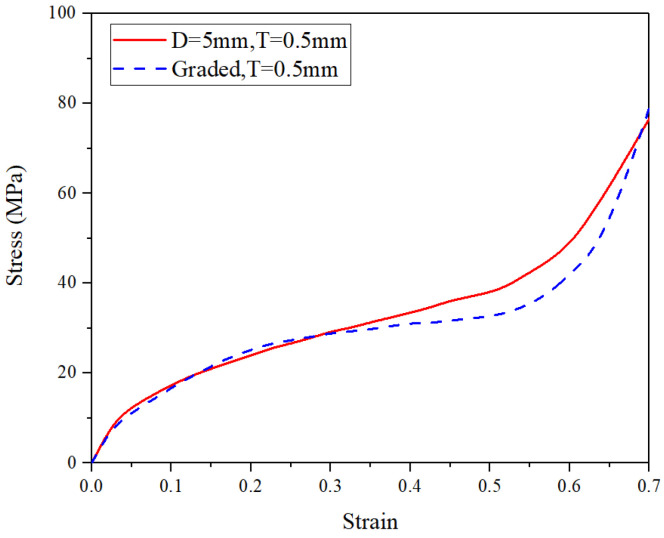
Stress–strain curves of the CCCFs with the same wall thickness and a different pore arrangement (i.e., the uniform and graded foam pores).

By using the FE simulations, the effects of pore diameter *D*, wall thickness *t*, and pore arrangement (i.e., either uniform or graded pore arrangement) on the compressive performance at the ambient temperature of 25 °C are discussed in this subsection. The compressive deformation of all kinds of CCCF models at the compressive strain of 0.5—including the initial undeformed configuration—are presented in Figure 14, Figure 15, Figure 16 and Figure 17, which compare the compressive stress–strain curves of all kinds of CCCF models: Figure 15 is for the uniform foams with the same wall thickness but a different pore diameter, Figure 16 for those of the uniform CCCFs with the same pore diameter but a different wall thickness, and Figure 17 is for the uniform and graded CCCFs with the same wall thickness.

It is concluded from Figure 12 that the compressive deformation of the cell walls is periodic and homogeneous for the uniform CCCFs (see Figure 12a–e), and the large von Mises stress concentrates on the cell walls along the compressive direction; meanwhile, for the graded CCCF (see Figure 12f), the collapse of cell walls in plastic deformation occurs first for the larger sphere pores, and subsequently for the smaller sphere pores layer by layer, which also corresponds to the nonperiodic and inhomogeneous distribution of the von Mises stress of the cell walls.

From Figure 15, it is found that the stress–strain curve of the uniform CCCFs with the same wall thickness will be increased as the pore diameter decreases; it can be seen from Figure 16 that the stress–strain curve of the uniform CCCFs with the same pore diameters increases with the increase of the cell wall thickness. In other words, both the decrease of pore diameter and the increase of cell wall thickness would improve the compressive performance of the uniform CCCFs. As for the CCCFs with the same wall thickness and a different pore arrangement (i.e., the uniform and graded foam pores) as shown in Figure 17, both of the two compressive stress–strain curves are very close, especially in the early stage before the compressive strain of 0.3; meanwhile, for the larger compressive strain exceeding 0.3, the stress–strain curve of the uniform CCCF rises rapidly, and that of the gradient CCCF still lasts in the plateau stage until the compressive strain up to 0.5. This leads to the larger onset strain of densification of the CCCF with gradient pore arrangement. It also explains the phenomenon in the previous experiments in which the plateau stress of the graded CCCF is lower than that of the uniform CCCF, but the energy absorption and energy absorption efficiency are higher for the graded CCCF.

## 4. Conclusions

The closed-cell copper foams (CCCFs) with prescribed sphere pores can be fabricated by powder metallurgy with the expanded polystyrene foams (EPS) spheres as the space holder. The feature of the microstructure was characterized, and the compressive behaviors of both uniform and graded CCCFs at different temperatures were experimentally and numerically studied. The main conclusions are summarized as follows:

(1) The fabrication process has the advantages of an adjustable pore structure, controllable porosity, but less meso-scale defects. The complete geometry of the sphere pores can be preserved in the foam samples after the sintering. There is less chemical residue of EPS in the as-fabricated copper foams, and no oxidation phenomenon occurred in the sintering process.
(2) A high temperature would greatly weaken the initial compressive modulus, plateau stress, and effective energy absorption for both uniform and graded CCCFs; meanwhile, the onset strain of densification and the maximum energy absorption efficiency are less sensitive to the temperature, especially for the graded CCCFs.
(3) In terms of energy absorption, the graded CCCF are superior to the uniform CCCF in both effective energy absorption and energy absorption efficiency. The maximum energy absorption efficiencies of graded CCCFs at different temperatures are all beyond 80%.
(4) Finite element simulations based on the ideal sphere foam model could basically mimic the compressive performance of the CCCF samples. It is numerically found that the dominated plastic deformation and stress distribution are periodic and homogenous for uniform CCCF, but both inhomogeneous for the graded one. The decrease of pore diameter and the increase of cell wall thickness could both improve the compressive performance of the CCCFs.

Present FE models only consisted of the mesoscopic sphere pores, rather than the microporous flaws induced by pressureless sintering and the surface damages caused by wire-cut electric discharge machining. In further work, the multi-scale numerical method will be developed to further refine the FE foam models and more accurately mimic compressive performance of the closed-cell copper foams.

## Figures and Tables

**Table 1:** Compressive properties of the uniform and graded CCCFs.

Type	Relative Density	Temperature (°C)	Initial Compressive Modulus of (MPa)	Onset Strain of Densification	Plateau Stress (MPa)	Effective Energy Absorption (MJ·m^−3^)	The Maximum Energy Absorption Efficiency (%)
Uniform	0.42	25	1462	0.29	25.48	5.84	79.66
		300	665	0.17	17.74	2.31	76.87
		500	266	0.14	10.71	1.15	76.48
Graded	0.37	25	1285	0.40	21.47	7.45	86.73
		300	332	0.37	14.62	4.85	89.71
		500	161	0.41	10.20	3.67	87.81
